# Electronic spectroscopy of cationic adamantane clusters and dehydrogenated adamantane in helium droplets†

**DOI:** 10.1039/d2cp03523e

**Published:** 2022-09-16

**Authors:** Miriam Kappe, Arne Schiller, Serge A. Krasnokutski, Milan Ončák, Paul Scheier, Ethan M. Cunningham

**Affiliations:** Institut für Ionenphysik und Angewandte Physik, Universität Innsbruck, Technikerstraße 25 6020 Innsbruck Austria milan.oncak@uibk.ac.at ethan.cunningham@uibk.ac.at; Laboratory Astrophysics Group of the MPI for Astronomy at the University of Jena, Helmholtzweg 3 D-07743 Jena Germany

## Abstract

We report the first helium-tagged electronic spectra of cationic adamantane clusters, along with its singly, doubly, and triply dehydrogenated analogues embedded in helium droplets. Absorption spectra were measured by recording the evaporation of helium atoms as a function of laser wavelength in the range of 300–2150 nm. Experimental spectra are coupled with simulated spectra obtained from quantum chemical calculations. The spectrum of cationic adamantane agrees with the electronic photodissociation spectrum measured previously, with an additional low-energy absorption at around 1000 nm. The spectra of the dehydrogenated molecules present broad absorptions exclusively in the high-energy region (300–600 nm). For the higher order adamantane dimer and trimer ions, strong absorptions are observed in the low-energy region (900–2150 nm), rationalised by transitions delocalised over two adamantane units.

## Abstract 2

We report the first helium-tagged electronic spectra of cationic adamantane clusters, along with its singly, doubly, and triply dehydrogenated analogues embedded in helium droplets.

## Graphical abstract

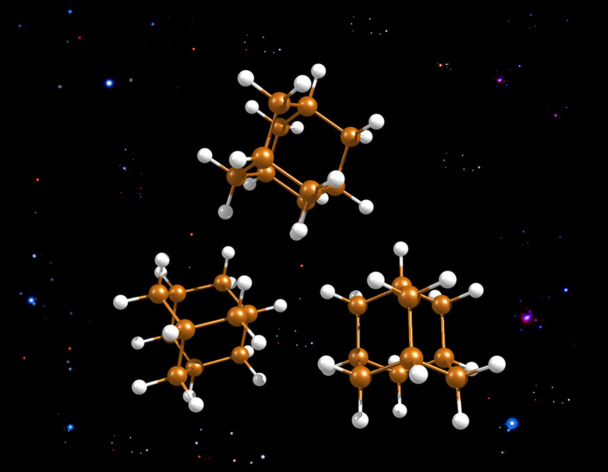

## Introduction

Diamondoids are classed as cage-like saturated rigid hydrocarbons, which have a diamond-like structure. Adamantane (C_10_H_16_; Adam) is the smallest and simplest diamondoid, whose C atoms adopt a diamond crystal lattice structure.^1–4^ Due to its structure, adamantane shows unique thermal stability and has become a popular hydrocarbon for a wide range of applications such as material and polymer science, molecular electronics, biomedical sciences, and chemical synthesis.^5–9^ Diamondoids are also expected as viable candidates as carriers for diffuse interstellar bands (DIBs),^10–17^ as their structural and thermal stability make them more able to withstand the harsher conditions in interstellar environments. As such, spectroscopic signatures of such compounds are required to compare with astronomical observations.^16^

Laboratory infrared spectroscopic measurements of diamondoids were compared with unidentified infrared emission bands present in the spectra of young stars with circumstellar disks, presenting noteworthy similarity.^18,19^ Diamondoids possess (relatively) low ionisation energies, thus the radical cations of these molecules were also suggested to be present in significant abundance, contributing to DIB spectra.^20–23^ The first cationic species identified as a DIB carrier was C_60_^+^, characterised *via* cryogenic ion trap spectroscopy of helium-tagged C_60_^+^.^16,24^ Laboratory-based molecular spectroscopy measurements are first needed to confirm the assignment of such molecules to DIBs. Early efforts include solid crystalline and solution phase infrared spectroscopy,^25–27^ and gas-phase emission infrared spectroscopy.^19,27^ Later, Dopfer and coworkers revealed significant evidence of Jahn–Teller distortion of cationic adamantane, C_10_H_16_^+^, using helium-tagging infrared photodissociation spectroscopy.^28^ Photoelectron total-ion-yield spectroscopy has also been employed by many groups to harvest electronic spectra of C_10_H_16_^+^, revealing experimentally derived ionisation energies.^29–31^ Time-dependent density functional theory (TDDFT) calculations were also performed by Steglich *et al.*,^21^ and later by Xiong and Saalfrank^32^ to yield the vertical absorption bands of C_10_H_16_^+^. To aid these assignments, infrared multiple-photon dissociation and threshold photoelectron spectroscopy measurements were carried out to explore electron ionisation and photodissociation products.^23,33^

Recently, Crandall *et al.* reported the optical spectrum of C_10_H_16_^+^ in the ultraviolet (UV) and near-infrared region employing electronic photodissociation spectroscopy.^17^ Even at cryogenic temperatures (20 K) the electronic transitions presented a broad spectrum, attributed to short lifetimes of excited states. The main photofragmentation channels were H loss at low energies and C_3_H_7_ loss at higher energies. The current study aims to investigate higher-order diamondoids, along with dehydrogenated analogues of C_10_H_16_^+^, employing helium-tagged electronic spectroscopy. This experimental method ensures a low-temperature environment for the aforementioned complexes and has been employed previously to directly compare with astrophysical observations.^16,34–38^

## Experimental and theory

Superfluid helium nanodroplets (HNDs) are formed *via* supersonic expansion of helium gas (2.9–3.1 MPa, 99.9999% purity) through a cooled nozzle (8.9–9.1 K, orifice diameter 5 μm) resulting in HNDs with a temperature of 0.37 K and mean droplet size of a few million He atoms.^39^ After passing through a skimmer (0.8 mm diameter) the HNDs are ionised by electron impact (electron energy 43 eV) leading to the formation of multiply charged droplets. The average charge state is estimated to be around +10 *e*.^40^ The highly charged droplet beam crosses a pick-up (PU) chamber where the droplets are doped with gas-phase adamantane. The adamantane powder (Sigma-Aldrich, 99% purity) sublimates at room temperature from a reservoir connected *via* a short gas line to the PU chamber. After pickup into a highly charged droplet, the first dopant which approaches a charge centre is ionised *via* charge transfer. The large difference in ionization energies of the He_*n*_^+^ charge centres and the dopants makes this process very exothermic, leading to fragmentation of the ionised dopant species. Pickup of multiple dopants leads to the growth of multiple, singly-charged dopant clusters, as the charges in helium droplets are separated and serve as nucleation centres for the growth of dopant clusters. In order to extract the dopant cluster ions from the large helium droplets, we collide them with a polished stainless-steel surface at a normal incidence angle. Most of the charge centres are recoiled backwards by reflecting helium or shock fronts. Most ions remain solvated by a small number of helium atoms, typically small enough to be accessible by mass spectrometry. Weak electrostatic fields are utilised to extract these ions from the collision region and guide them into a time-of-flight mass spectrometer (Tofwerk AG model HTOF), which is set orthogonal to the HND beam direction. Mass spectra were obtained with a resolution of approximately 1400*m*/Δ*m*. A tunable pulsed laser (EKSPLA NT242, up to 450 μJ pulse energy, laser bandwidth < 3.5 cm^−1^) is utilized to perform action spectroscopy upon electronic excitation of the helium-tagged title complexes. The laser was calibrated using a wavelength meter (SHR High-Resolution Wide-Range Wavelength Meter). The mass spectrometer operates at a frequency of 10 kHz, while the laser has a repetition rate of 1 kHz. The synchronization of the laser pulse with every tenth extraction pulse of the TOF allows for a direct comparison of mass spectra with and without laser irradiation. Absorption of a resonant photon leads to the evaporation of the weakly bound He atoms. Using the adamantane cation ion signal as an example, the signature for photon absorption can be monitored as a depletion of the corresponding He_*n*_Adam^+^ ion signal and a concomitant increase of the photoproduct Adam^+^ ion signal, respectively. Absorption spectra were recorded in several separate laser scans from 300 to 600 nm and 900 to 2150 nm for (Adam)^+^, the singly, C_10_H_15_^+^, and doubly dehydrogenated adamantane cation, C_10_H_14_^+^, and from 410 to 600 nm and 900 to 2150 nm for the adamantane dimer, (Adam)_2_^+^, and trimer cation, (Adam)_3_^+^. For the triply dehydrogenated adamantane cation, C_10_H_13_^+^, spectra were recorded in the 900 to 2150 nm region. The excitation wavelength used for each spectrum was scanned in step sizes of 0.5, 1.0, or 2.0 nm. All shown absorption spectra have been corrected for differences in photon densities at different laser wavelengths, assuming a direct correlation between laser power and the change in ion signal.

Structures of adamantane, its clusters, and dehydrogenated adamantane cations were optimised at the B3LYP/aug-cc-pVDZ level using the D3 dispersion correction as suggested by Grimme *et al.*^41^ For dehydrogenated adamantane cations, C_10_H_13–15_^+^, we chose all symmetrically inequivalent structures arising from the removal of hydrogen atoms, optimised them at the PBE+D3/6-31g* level and subsequently at the B3LYP+D3/aug-cc-pVDZ level. This led to 2, 7, and 20 unique structures for C_10_H_15_^+^, C_10_H_14_^+^, and C_10_H_13_^+^, respectively. We denote the isomers according to hydrogen atoms that were removed either from CH or CH_2_ groups, *e.g.*, a structure with two hydrogen atoms removed from the CH_2_(A) group and one hydrogen from the CH(A) group is classified as “CH_A_,CH_2A,A_”. To obtain more reliable reaction energies for dehydrogenation, we performed single-point recalculation on the coupled cluster singles and doubles with non-iteratively included triplets (CCSD(T)) level of theory. Reported energies were zero-point energy corrected, only vertical ionisation energies were not. Charge analysis was performed using the CHELPG scheme.^42^

For simplicity, electronic transitions were evaluated for ions without adsorbed helium atoms. Excited states were calculated using the Equation of Motion CCSD (EOMCCSD) method. For adamantane dimer and trimer cations, we used time-dependent density functional theory (TDDFT) employing the BHandHLYP functional that performs well for the adamantane cation and C_10_H_15_^+^ compared to the EOMCCSD method (see ESI,† Tables S1–S3). Electronic transitions up to energies of 6.2 eV (200 nm) were considered. The excitation character was described using the natural transition orbital scheme at the TDDFT level.^43^ The widths of the absorption bands were modelled using linearised reflection principle approximation, employing only single-point information on vibrations in the ground state and forces in excited states.^44–47^ Within this approximation, the spectral vibrational resolution is neglected. Spectra modelling was performed using the B3LYP+D3/aug-cc-pVDZ method for the ground electronic state along with EOMCCSD/aug-cc-pVDZ excitation energies and oscillator strengths, and BHandHLYP/aug-cc-pVDZ forces in the excited state; for adamantane dimer and trimer cations, BHandHLYP/aug-cc-pVDZ was used also for excitation energies and oscillator strengths. All electronic structure calculations were performed using the Gaussian software.^48^

## Results and discussion

**Fig. 1:**
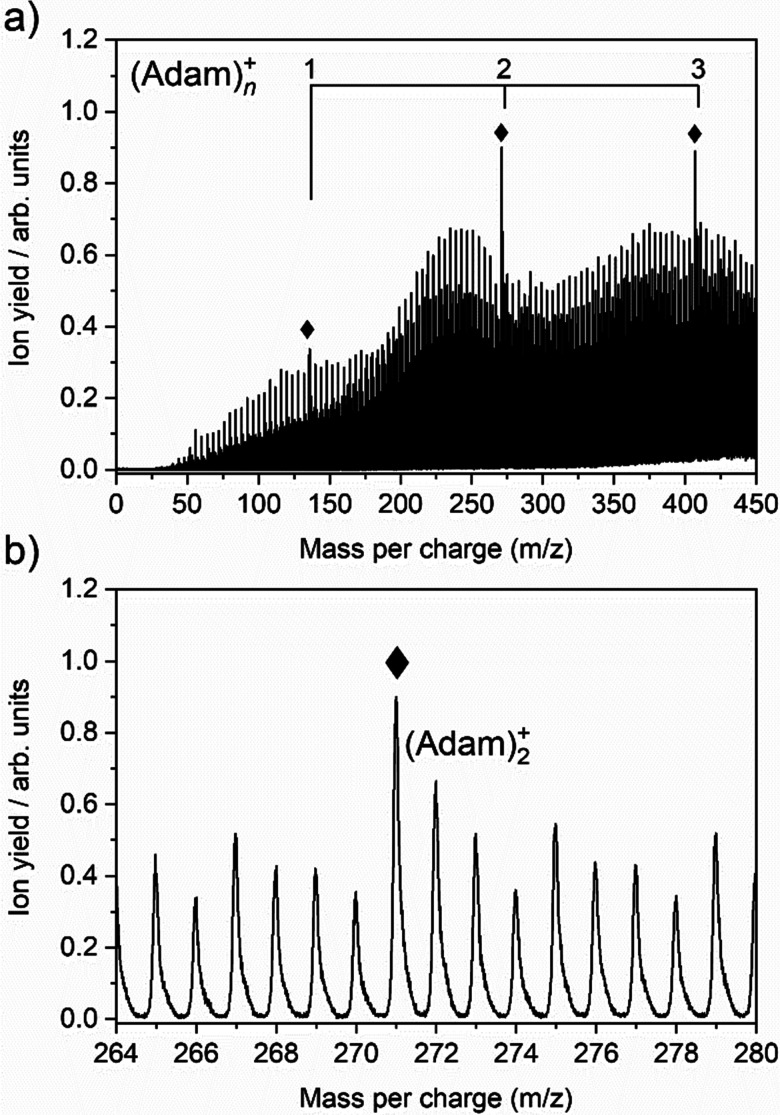
(a) Time-of-flight mass spectrum of helium nanodroplets doped with adamantane, showing cationic adamantane clusters, (Adam)_*n*_^+^, *n* = 1–3 (136, 272, and 408 *m*/*z*). Ion signals corresponding to the dehydrogenated analogues are also highlighted (♦). (b) Inlet between 264–280 mass units to highlight the cationic adamantane dimer and its dehydrogenated analogue at 272 and 271 *m*/*z*, respectively.

The cluster formation of adamantane cations within helium droplets has been explored previously by our group,^49–51^ employing time-of-flight mass spectrometry. Fig. 1a shows a time-of-flight mass spectrum of helium nanodroplets doped with adamantane up to 450 mass units. Prominent features include cationic adamantane clusters, (Adam)_*n*_^+^, *n* = 1–3 (136, 272, and 408 *m*/*z*) along with their dehydrogenated analogues, highlighted with diamonds. In the case of the monomer, (Adam)^+^, its ion signal is slightly more intense than the dehydrogenated analogue, whereas the reverse is true for the dimer and trimer adamantane ion signals. Fig. 1b shows that the adamantane dimer (272 mass units) is weaker than its dehydrogenated analogue (271 mass units). The strong preference for dehydrogenation was rationalised previously^49^ by considering the following reaction:

1 He^+^ + C_10_H_16_ → C_10_H_15_^+^ + H + He

Reaction 1 is exothermic by 14.0 eV, given the ionisation energy of helium is 24.6 eV and the appearance energy of C_10_H_15_^+^ is 10.6 eV.^52^ The increased ion signal at 271 mass units can be explained by the very weak binding energy of the H atom after ionization of the adamantane molecule, calculated as 1.12 eV (Table 1). At the same time, H loss is not that efficient when clusters are ionized since the binding energy between the adamantane molecule and adamantane ion is around half that of H loss. Therefore, in order for these weakly bound complexes to remain intact, most of the excess energy during the ionization must be dissipated by the helium droplet.

**Table 1:** Reaction energies (in eV) calculated at the B3LYP+D3/aug-cc-pVDZ and CCSD(T)/aug-cc-pVDZ//B3LYP+D3/aug-c-pVDZ levels of theory (denoted as B3LYP+D3 and CCSD(T)//B3LYP+D3, respectively), considering the most stable isomers as calculated at the B3LYP+D3/aug-cc-pVDZ level. Vertical ionization energies of C_10_H_16_ are given in parentheses

Reaction	B3LYP+D3	CCSD(T)//B3LYP+D3
C_10_H_16_ → C_10_H_16_^+^ + e^−^	8.81 (9.41)	9.19 (9.89)
C_10_H_16_^+^ → C_10_H_15_^+^ + H	1.50	1.12
C_10_H_15_^+^ → C_10_H_14_^+^ + H	3.80	3.73
C_10_H_16_^+^ → C_10_H_14_^+^ + H_2_	0.92	0.60
C_10_H_15_^+^ → C_10_H_13_^+^ + H_2_	1.72	1.61
C_10_H_14_^+^ → C_10_H_13_^+^ + H	2.29	2.13
(C_10_H_16_)_2_^+^ → C_10_H_16_^+^ + C_10_H_16_	0.67	—
(C_10_H_16_)_3_^+^ → (C_10_H_16_)_2_^+^ + C_10_H_16_	0.53	—

**Fig. 2:**
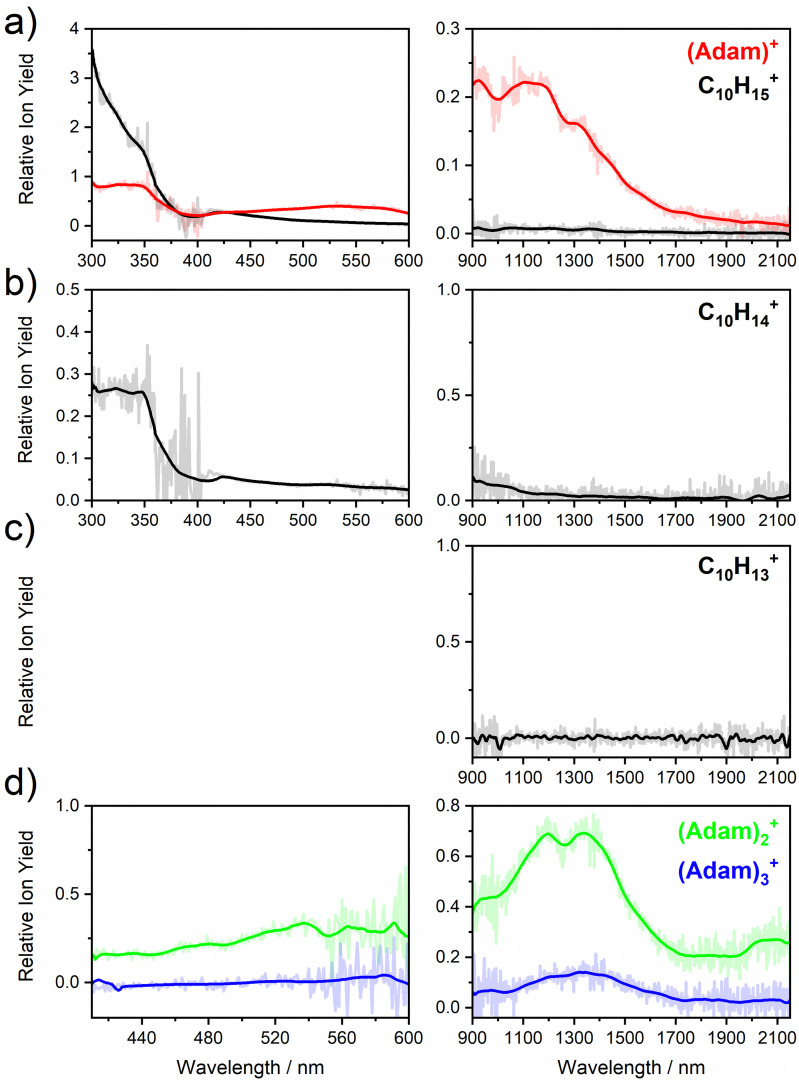
Absorption spectra of (a) (Adam)^+^ and its dehydrogenated analogue, (b) the doubly dehydrogenated analogue, C_10_H_14_^+^, (c) the triply dehydrogenated analogue, C_10_H_13_^+^, and (d) the adamantane dimer, and trimer cation, (Adam)_2_^+^ and (Adam)_3_^+^. In each case, the spectrum is recorded by monitoring the increase in ion signal of the “bare” ion mass.

**Fig. 3:**
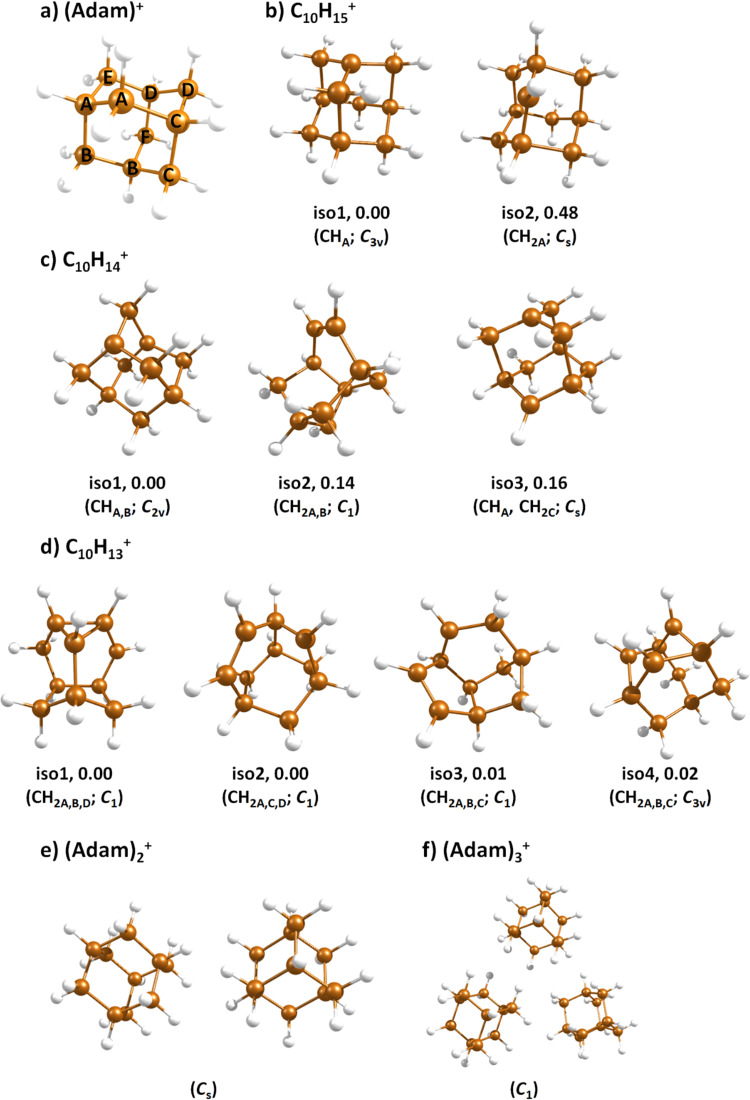
Selected isomers of (a) (Adam)^+^, (b) C_10_H_15_^+^, (c) C_10_H_14_^+^, (d) C_10_H_13_^+^, (e) (Adam)_2_^+^, and (f) (Adam)_3_^+^ optimised at the B3LYP+D3/aug-cc-pVDZ level of theory along with the relative isomer energy in eV and symmetry point group. In (b–d), hydrogen atoms removed from CH and CH_2_ groups of (Adam)^+^ are denoted according to atom nomenclature shown in (a).

**Fig. 4:**
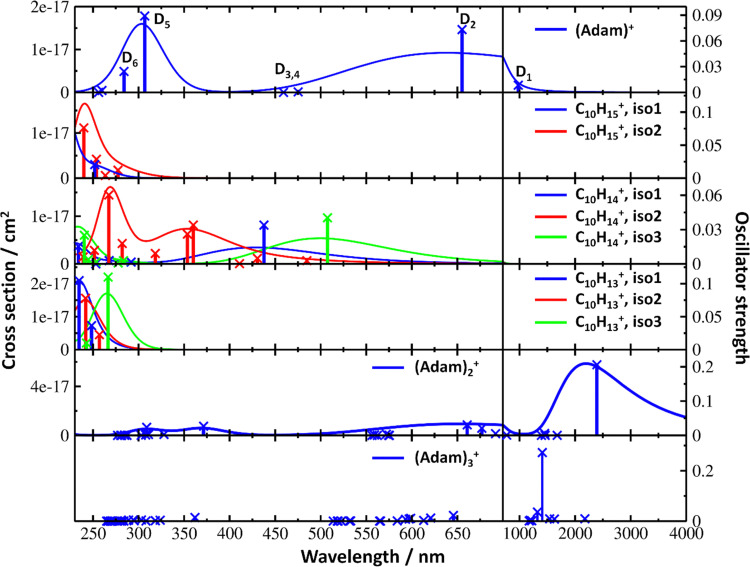
Simulated electronic absorption spectra of structures shown in Fig. 3. Transition energies and oscillator strengths were calculated at the EOMCCSD/aug-cc-pVDZ level except for (Adam)_2_^+^ and (Adam)_3_^+^ which were calculated at the TD-BHandHLYP/aug-cc-pVDZ level. Forces in the excited states were modelled at the TD-BHandHLYP/aug-cc-pVDZ level, structures were optimised at the B3LYP+D3/aug-cc-pVDZ level. Transitions with their oscillator strength (denoted as bars, transition positions are shown by crosses) and spectra modelled within the linearised reflection principle (lines, no spectrum was modelled for (Adam)_3_^+^) are shown.

**Fig. 5:**
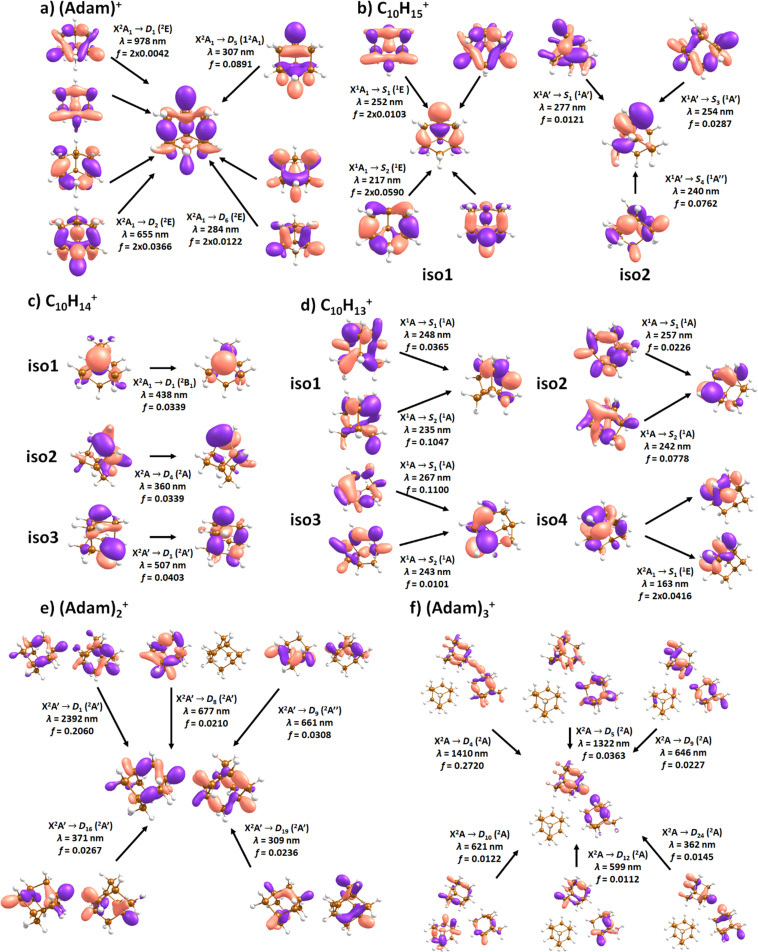
Character of selected electronic transitions in isomers shown in Fig. 3. Calculated at the EOMCCSD/aug-cc-pVDZ level for (a–d) and TD-BHandHLYP/aug-cc-pVDZ for (e and f). Structures were optimised at the B3LYP+D3/aug-cc-pVDZ level, natural transition orbitals at the TD-BHandHLYP/aug-cc-pVDZ level are shown.

Fig. 2a shows the electronic absorption spectra of (Adam)^+^ (red) and its dehydrogenated analogue, C_10_H_15_^+^ (black), measured from 300–600 and 900–2150 nm. In the 600–900 nm region, the laser power is significantly reduced, causing the signal-to-noise ratio to rapidly decrease. Thus, the following discussion will focus only on the regions covered in Fig. 2. The electronic spectra in Fig. 2 are produced from dissociation of many helium-tagged complexes, so present broader bands when compared to astronomical observations. The spectra discussed in the present study are therefore used only to gain an overall understanding of the molecular spectra. Calculated structures of investigated ions are included in Fig. 3, modelled absorption spectra in Fig. 4, and the character of excited states is depicted in Fig. 5.

### C_10_H_16_^+^: (Adam)^+^

After the adamantane molecule is ionized, symmetry lowering is observed from tetrahedral symmetry, *T*_d_, of the neutral molecule to *C*_3v_ of the cation, with the ^2^A_1_ ground state (the electron is removed from an a_1_ orbital). The vertical ionisation energy is calculated at the CCSD(T)/aug-cc-pVDZ//B3LYP+D3/aug-cc-pVDZ level as 9.89 eV, the adiabatic ionisation energy is evaluated as 9.19 eV. These are close to the experimental vertical and adiabatic ionisation energies of 9.44–9.75 eV and 9.23–9.40 eV.^29,30,53^

The spectrum of (Adam)^+^ in Fig. 2a is qualitatively similar to the gas-phase spectrum of mass-selected cationic adamantane studied *via* photodissociation spectroscopy by Dopfer and coworkers.^17^ In their study, the experimentally observed bands were assigned to calculated vertical excitation energies of (Adam)^+^, computed at the TDDFT level. Our calculations on electronic states in (Adam)^+^ are in full agreement with those published previously.^32^ All electronic excitations in the investigated range result from the excitation of an electron into the singly occupied molecular orbital (SOMO) of a_1_ irreducible representation (Fig. 5a).

The difference to the study by Dopfer *et al.*^17^ is the appearance, at low energies, of the optically allowed D_1_ (^2^E) transition, which was unobserved in their study. The hydrogen dissociation requires 1.12 eV (Table 1), which could be a reason why the band was not observed in previous experiments, along with low intensity of the transition into D_1_ compared to the one into D_2_. This band has a maximum at 1104 nm or 1.12 eV (Fig. 2a), its position is calculated at 978 nm (1.27 eV) at the EOMCCSD/aug-cc-pVDZ level. It should be noted that the spectrum acquired in the present study results from helium evaporation, leading to an enhancement in the bare (Adam)^+^ signal. Thus, lower energy region bands, such as the D_1_ (^2^E) band, can be detected. The photon energy at this wavelength is not sufficient to fragment the (Adam)^+^. Therefore, photodissociation spectroscopy, which relies on the detection of photoproducts, cannot reveal this band. The experimentally observed band shows no vibrational progression, hinting towards a fast reconstruction of the D_0_ state, without any minimum on the D_1_ surface. The width of the band is calculated as ∼0.9 eV, in reasonable agreement with the experiment.

The broad feature between 450–600 nm is assigned to the second electronically excited, twofold degenerate doublet state D_2_ of ^2^E irreducible representation. The excitation energy in the minimum structure is calculated as 1.89 eV (655 nm, oscillator strength *f* = 0.037). Previous calculations by Xiong and Saalfrank^32^ using a time-dependent correlation function approach at the TD-B3LYP level predicted a width of 0.6 eV for this transition, rationalised by significant Franck–Condon activity due to pronounced geometry changes involved in D_2_ excitation. The linearised reflection principle approximation employed here also predicts a broad (∼0.9 eV) band, however is in good agreement with the D_2_ (^2^E) band in the present study, and the study by Dopfer *et al.*^17^ The calculated width in fact hides the band arising from the transition into the D_1_ (^2^E) state (Fig. 4).

The calculated oscillator strength for excitation into D_3_ (^2^E) is very low (*f* = 3 × 10^−4^), and the transition into D_4_ (^2^A_2_) is symmetrically forbidden. These two transitions thus do not contribute to the measured spectrum. The band at *ca.* 325 nm is in agreement with the assignment by Dopfer *et al.*^17^*i.e.*, the transitions to D_5_ (^2^A_1_) and D_6_ (^2^E) states, here calculated to lie at 307 and 284 nm, respectively. The calculated onset of the band at ∼375 nm in Fig. 4 is in good agreement with the experimental spectrum in Fig. 2a.

For each band, no vibrational fine structure is observed in the experiment. In contrast, the high energy region (>3.5 eV) bands showed vibrational structure in the study by Dopfer,^17^ attributed to a progression in the *ν*_1_ and *ν*_2_ fundamental modes, and combination band, *ν*_1_ + *ν*_2_. Relatively smaller progressions were attributed to lifetime broadening of single vibronic transitions. The absence of vibrational structure in the present study could be due to lifetime broadening, whereby the evaporation of helium atoms from (Adam)^+^ is a much faster process, when compared to direct photodissociation, leading to spectral broadening. Another explanation could be that the large number of helium atoms attached, each with slight variations in absorption energy, lead to smearing of the vibrational fine structure.

### C_10_H_13–15_^+^

When a hydrogen dissociates from the adamantane cation, producing C_10_H_15_^+^, two different isomers could emerge (Fig. 3b). In isomer 1, the hydrogen leaves a CH group, retaining *C*_3v_ symmetry of (Adam)^+^. Isomer 2, with hydrogen dissociating from a CH_2_ group, is calculated to possess *C*_s_ symmetry and is predicted to be less stable by 0.48 eV than isomer 1. The H loss from C_10_H_16_^+^ requires only ∼1.12 eV (Table 1).

Given that the electronic absorption spectrum of (Adam)^+^ in the present study (Fig. 2a) is in good agreement with previous calculations and experimental studies,^17,32^ this can be used as a calibration spectrum, for which the spectra of the other ions in this study can be compared to. The spectra of the dehydrogenated analogue of cationic adamantane, C_10_H_15_^+^, is shown in Fig. 2a (black). To the best of our knowledge, this is the first electronic spectrum of C_10_H_15_^+^.

The first difference between C_10_H_15_^+^ and (Adam)^+^ to note is in the 900–2150 nm region; no band is observed here for C_10_H_15_^+^. Indeed, our calculations predict no transitions in this wavelength region, as C_10_H_15_^+^ is a closed-shell species of singlet spin multiplicity. Both isomers present no transitions above 280 nm. Indeed, the experimental spectrum shows an onset of an intense band starting at *ca.* 375 nm with the band maximum not reached. Our simplified bandwidth modelling within linearised reflection principle predicts that the strong onset observed experimentally is most likely the onset of the predicted electronic transitions. The similar shape of the (Adam)^+^ and C_10_H_15_^+^ spectra in this region also hints that some C_10_H_15_^+^ ions might be produced from (Adam)^+^ through hydrogen dissociation. However, this is most likely a minor contribution in this high-energy region, based on the study by Dopfer.^17^

The electronic transitions in the region of interest proceed into the lowest unoccupied molecular orbital (LUMO), which is composed of the 2p orbital of the carbon atom from which the hydrogen was removed (Fig. 5b), the LUMO of isomer 1 resembles the SOMO of (Adam)^+^. Compared to previous calculations on C_10_H_15_^+^ at the TD-B3LYP/TZVP level,^32^ our calculated excitation energies lie considerably higher in energy, by ∼0.6 eV. According to our benchmark calculations, the TD-B3LYP approach provides consistently lower excitation energies for this structure compared to other DFT functionals and the EOMCCSD method (see Tables S2 and S3, ESI†).

The (putative) most stable isomer of C_10_H_14_^+^ is formed by dehydrogenating two CH units and possesses *C*_2v_ symmetry, isomer 1, two further isomers lie close in energy (Fig. 3c, see the ESI† for all located isomers). Dissociation of two H atoms from Adam^+^ requires 4.85 eV (0.60 eV if they dissociate as molecular hydrogen, H_2_). As C_10_H_14_^+^ has doublet spin multiplicity, low-lying excitations are present again. In the experimental spectrum shown in Fig. 2b, we observe a weak onset at ∼1000 nm, a broad band at 400–600 nm, and a strong absorption at 300–350 nm. All these features are reproduced in our calculations if we assume the presence of several isomers (Fig. 4), with a band of the lowest-lying isomer peaking at ∼430 nm, isomer 2 absorbing strongly below 400 nm and the first band of isomer 3 absorbing up to 900 nm. Alternatively, the experimental absorption below 350 nm might arise through absorption of C_10_H_15_^+^ following H loss that requires ∼3.7 eV, corresponding to ∼340 nm.

For C_10_H_13_^+^, 20 local minima were found. Four isomers are found to lie within 0.02 eV, with the common motif of hydrogen atoms leaving three different CH_2_ groups (Fig. 3d). In the most stable isomer, 1, a ring of three CH groups is formed, with C–C distances of 1.45, 1.61, and 1.66 Å. In the measured electronic spectra, Fig. 2c, we observe no intensity in the 900–2150 nm region. As these species are closed-shell singlets, no low-lying electronic transitions are to be expected. Accordingly, calculated absorption spectra show intensity only below 325 nm (Fig. 4), and in extreme cases, such as isomer 4, below 165 nm. The experimentally observed spectra thus cannot be explained by direct absorption of C_10_H_13_^+^ unless higher-lying isomers are present. Unfortunately, no meaningful spectrum could be measured in the 300–600 nm region, so cannot be compared to the simulated spectrum (Fig. 4) in this region.

### (Adam)_2,3_^+^

The adamantane dimer cation, (Adam)_2_^+^, was optimised to a structure of *C*_s_ symmetry (Fig. 3e), with an almost equal distribution of charge according to the CHELPG method (0.52 : 0.48). The binding energy with respect to a neutral adamantane molecule and (Adam)^+^ is calculated as 0.67 eV (Table 1).

The electronic absorption spectrum of (Adam)_2_^+^ is shown in Fig. 2d. Four main features are observed in the 900–2150 nm region; a weak feature at 2064 nm, two strong features at 1334 and 1190 nm, and a shoulder at 962 nm. Apart from the weak feature at 2064 nm, the positions of these features are similar to those observed in the spectrum of (Adam)^+^ (features at 924, 1122, and 1308 nm, Fig. 2a).

According to our calculations, a considerable change in the absorption spectrum takes place compared to that of (Adam)^+^, see Fig. 4. There is one very intense absorption calculated at 0.52 eV (*ca.* 2400 nm), the remaining transitions have considerably lower oscillator strength, forming two broad absorptions at 300–400 and 500–900 nm. This pattern is seen in calculations in both TD-BHandHLYP and TD-CAMB3LYP methods, the bright transition at low excitation energy is observed also at the EOMCCSD level (see Table S4, ESI†). As seen already for (Adam)^+^, all electronic transitions up to 6 eV proceed into the SOMO orbital.

The experimental band at 2064 nm can be assigned as the predicted low-lying bright transition. The calculated width is considerably larger than the experimental band, the plateau seen in the experiment could however come from a sum of bands at ∼1300 nm and ∼2000 nm, making an accurate width calculation difficult. The band at ∼1300 nm is not seen in the calculations. A possible explanation is that the (Adam)_2_^+^ ion is formed from higher oligomers (Adam)_*n*_^+^, *n* > 2, at these wavelengths. As we show below, (Adam)_3_^+^ has a broad absorption in the 1000–1600 nm region, matching the observed band in the spectrum of (Adam)_2_^+^. The calculated dissociation energy of (Adam)_3_^+^ to (Adam)_2_^+^ + (Adam) is 0.53 eV, corresponding to ∼2300 nm. The experimentally observed broad peak at 400–600 nm is in good agreement with several electronic transitions predicted by our calculations.

In the optimised structure of (Adam)_3_^+^, the adamantane moieties form a triangle (Fig. 3f). The charge distribution of 0.42 : 0.40 : 0.18 on the adamantane units suggests that the system might be viewed as (Adam)_2_^+^ with an almost neutral adamantane molecule attached. The measured spectrum of (Adam)_3_^+^ in Fig. 2d shows a broad band in the 1000–1600 nm region and a less intense band at 540–600 nm. The calculations predict again only transitions into the SOMO orbital, the electron might however be excited from each adamantane unit, depending on the given electronic state (Fig. 5f). The modelled spectra show several intense transitions at ∼1500 nm and lower-intensity transitions in the 550–700 nm and 270–400 nm regions, Fig. 4. The experimental spectrum is very similar to that of (Adam)_2_^+^, only the lowest-energy transitions are slightly blue-shifted. Overall, the calculated transitions are in good agreement with the measured spectrum. However, part of the signal might again come from higher oligomers.

The performed measurements demonstrated that at least small diamondoids are not promising candidates for the DIB carriers. In the neutral state, they absorb in the UV range, outside the position where the DIBs are observed. After ionization, however, they become very unstable; the loss of an H atom requires only 1.12 eV, which can be provided by a 1110 nm photon. Also, considering the extremely wide absorption bands of adamantane cations that cover almost the entire visible and NIR regions of the spectrum, we can conclude that (Adam)^+^ is unstable in the regions of the interstellar medium (ISM) where the DIBs are commonly detected. (Adam)^+^ should decay very quickly to C_10_H_14_^+^ after absorption of a photon in the visible wavelength region. Therefore, adamantane cations should mainly exist in the dehydrogenated form in the ISM. Our spectroscopy study shows that this form of adamantane (C_10_H_14_^+^) is also not a promising carrier of DIBs. Similar to the parent, (Adam)^+^, it shows very broad absorption bands, far exceeding the width of the DIBs. Moreover, the absorption of C_10_H_14_^+^ also lies outside of the DIB range. Even more important to consider is the loss of C_3_H_7_ at high photon energies. This leads to the destruction of the carbon cage of the molecule, which likely excludes the bottom-up formation route for diamondoids in the gas phase in the ISM. However, larger diamondoids should demonstrate better photostability, so are more likely to survive in the ISM. Their presence in the ISM can be implied based on the presence of interstellar nanodiamonds in meteorites.^54^ Therefore, the study of photostability and spectroscopy of larger diamondoids is important for understanding the properties of this entire class of molecules.

## Conclusions

We have measured electronic action spectra of the adamantane cation, its three dehydrogenated forms C_10_H_15_^+^, C_10_H_14_^+^, and C_10_H_13_^+^ along with the dimer and trimer cations in the 300–2150 nm wavelength range. Our quantum chemical calculations are able to reproduce all main features in the spectra, including spectral onsets. The monomer spectrum is in agreement with those measured previously;^17^ additionally, we revealed a new, low-energy absorption reaching a plateau at ∼1000 nm. Dehydrogenated structures show broad absorptions in the 300–600 nm region and are most probably partially formed by dehydrogenation of ions with more hydrogen atoms. We suggest that several isomers with very similar energies might co-exist for C_10_H_14_^+^ and C_10_H_13_^+^. For adamantane dimer and trimer cations, strong absorption bands in the low-energy region are observed, corresponding to transitions delocalised over two adamantane units. For (Adam)_2_^+^, the charge is delocalised almost equally on both units, one adamantane moiety in (Adam)_3_^+^ is then mostly a spectator, although it is involved in some electronic transitions. Our study showed that adamantane and its dehydrogenated cations are not the carriers of DIBs. It also showed a very low photostability of these molecules, excluding its survival in the regions of the ISM, where DIBs are detected. The study of large diamondoids is suggested for better understanding of interstellar diamond formation.

## Conflicts of interest

There are no conflicts to declare.

## Supplementary Material

CP-024-D2CP03523E-s001
