# Supplementary material for: Electronic spectroscopy of cationic adamantane clusters and dehydrogenated adamantane in helium droplets
Source: Phys Chem Chem Phys. 2022 Sep 16;24(38):23142–51. doi: 10.1039/d2cp03523e (PMC9533311; doi:10.1039/d2cp03523e)
Supplement: CP-024-D2CP03523E-s001 [file CP-024-D2CP03523E-s001.pdf]

Electronic Supplementary Material (ESI) for Physical Chemistry Chemical Physics.
This journal is © the Owner Societies 2022

Supporting Information

Electronic spectroscopy of cationic adamantane clusters and dehydrogenated adamantane in helium droplets

Miriam Kappe[1], Arne Schiller[1], Serge A. Krasnokutski[2], Milan Ončák[1],* Paul Scheier[1], and Ethan M. Cunningham[1]*

1. Institut für Ionenphysik und Angewandte Physik, Universität Innsbruck,

   Technikerstraße 25, 6020 Innsbruck, Austria

2. Laboratory Astrophysics Group of the MPI for Astronomy at the University of Jena,

   Helmholtzweg 3, D-07743 Jena, Germany

Corresponding authors: milan.oncak@uibk.ac.at, ethan.cunningham@uibk.ac.at

Table S1 – Excitation energies *E* (in eV) and oscillator strengths *f* in $(Adam)^+$ calculated using various methods in the structure optimized at the B3LYP+D3/aug-cc-pVDZ level.

state	EOMCCSD/ aug-cc-pVDZ		TD-BHandHLYP/ aug-cc-pVDZ		TD-CAMB3LYP/ aug-cc-pVDZ		TD-B3LYP/ aug-cc-pVDZ	
	E	*F*	*E*	*f*	*E*	*f*	*E*	*f*
E	1.27	0.0042	1.25	0.0063	1.16	0.0026	1.08	0.0009
E	1.89	0.0366	1.79	0.0311	1.80	0.0321	1.83	0.0293
E	2.61	0.0003	2.60	0.0003	2.44	0.0004	2.33	0.0010
A_2	2.70	0.0000	2.69	0.0000	2.49	0.0000	2.31	0.0000
A_1	4.04	0.0891	3.89	0.0799	3.83	0.0750	3.80	0.0615
E	4.37	0.0122	4.23	0.0101	4.04	0.0122	3.80	0.0128
E	4.77	0.0011	4.66	0.0012	4.46	0.0002	4.18	0.0000
A_2	4.84	0.0000	4.79	0.0000	4.55	0.0000	4.27	0.0000
E	6.25	0.0021	6.05	0.0017	5.79	0.0016	5.43	0.0014

Table S2 – Excitation energies *E* (in eV) and oscillator strengths *f* in $C_{10}H_{15}^+$, isomer 1, calculated using various methods in the structure optimized at the B3LYP+D3/aug-cc-pVDZ level.

state	EOMCCSD/ aug-cc-pVDZ		TD-BHandHLYP/ aug-cc-pVDZ		TD-BHandHLYP/ aug-cc-pVTZ		TD-CAMB3LYP/ aug-cc-pVDZ		TD-B3LYP/ aug-cc-pVDZ		TD-B3LYP/TZVP	
	E	*f*	*E*	*f*	*E*	*F*	*E*	*f*	*E*	*f*	*E*	*f*
E	4.92	0.0103	5.14	0.0069	5.15	0.0070	4.75	0.0041	4.31	0.0014	4.31	0.0013
E	5.72	0.0590	6.01	0.0447	6.01	0.0444	5.60	0.0453	5.17	0.0438	5.17	0.0452
A_2	6.32	0.0000	6.57	0.0000	6.57	0.0000	6.02	0.0000	5.56	0.0000	5.56	0.0000
E	6.57	0.0076	6.71	0.0116	6.70	0.0114	6.29	0.0101	5.89	0.0053	5.89	0.0055

Table S3 – Excitation energies *E* (in eV) and oscillator strengths *f* in $C_{10}H_{15}^+$, isomer 2, calculated using various methods in the structure optimized at the B3LYP+D3/aug-cc-pVDZ level.

state	EOMCCSD/ aug-cc-pVDZ		TD-BHandHLYP/ aug-cc-pVDZ		TD-BHandHLYP/ aug-cc-pVTZ		TD-CAMB3LYP/ aug-cc-pVDZ		TD-B3LYP/ aug-cc-pVDZ		TD-B3LYP/TZVP	
	E	*f*	*E*	*f*	*E*	*f*	*E*	*f*	*E*	*f*	*E*	*f*
A'	4.47	0.0121	4.68	0.0113	4.70	0.0113	4.23	0.0096	3.71	0.0076	3.71	0.0079
A"	4.71	0.0040	4.86	0.0033	4.88	0.0034	4.45	0.0005	3.90	0.0001	3.89	0.0001
A'	4.89	0.0287	5.06	0.0234	5.07	0.0234	4.62	0.0194	3.94	0.0135	3.94	0.0140
A"	5.17	0.0762	5.43	0.0551	5.43	0.0549	5.08	0.0528	4.67	0.0308	4.67	0.0304
A"	5.83	0.0006	6.06	0.0016	6.07	0.0015	5.54	0.0016	4.90	0.0107	4.90	0.0120
A'	5.86	0.0024	6.00	0.0020	6.00	0.0019	5.57	0.0024	5.05	0.0040	5.05	0.0042
A"	6.15	0.0002	6.27	0.0004	–	–	5.83	0.0000	5.31	0.0005	5.31	0.0005

Table S4 – Excitation energies *E* (in eV) and oscillator strengths *f* in $(Adam)_2^+$ calculated using various methods in the structure optimized at the B3LYP+D3/aug-cc-pVDZ level.

state	BHandHLYP/ 6-31g*		CAMB3LYP/ aug-cc-pVDZ		B3LYP/aug-cc-pVDZ		BHandHLYP/ aug-cc-pVDZ		EOMCCSD/ 6-31g*	
	E	*f*	*E*	*f*	*E*	*f*	*E*	*f*	*E*	*f*
A'	0.54	0.2098	0.49	0.2068	0.62	0.0003	0.52	0.2060	0.51	0.1799
A'	0.73	0.0002	0.70	0.0002	0.69	0.0207	0.74	0.0003	0.76	0.0039
A"	0.84	0.0002	0.79	0.0001	0.67	0.0001	0.85	0.0002	0.87	0.0003

A'	0.85	0.0064	0.82	0.0058	0.80	0.1627	0.85	0.0058	0.99	0.0015
A"	0.88	0.0001	0.83	0.0001	0.69	0.0000	0.89	0.0001	1.07	0.0000
A"	1.61	0.0006	1.60	0.0005	1.55	0.0000	1.61	0.0006	–	–
A'	1.79	0.0054	1.78	0.0037	1.68	0.0045	1.79	0.0050	–	–
A'	1.83	0.0229	1.83	0.0210	1.73	0.0197	1.83	0.0210	–	–
A"	1.88	0.0334	1.85	0.0284	1.78	0.0240	1.88	0.0308	–	–
A"	2.15	0.0000	2.06	0.0002	1.87	0.0005	2.15	0.0000	–	–
A"	2.15	0.0003	2.06	0.0003	1.91	0.0001	2.16	0.0002	–	–
A'	2.16	0.0034	2.09	0.0036	1.93	0.0031	2.17	0.0034	–	–
A'	2.20	0.0001	2.14	0.0001	1.98	0.0003	2.21	0.0000	–	–
A"	2.21	0.0012	2.12	0.0004	1.92	0.0000	2.22	0.0011	–	–
A"	2.22	0.0000	2.13	0.0018	1.98	0.0036	2.23	0.0000	–	–
A'	3.36	0.0275	3.27	0.0262	3.21	0.0247	3.34	0.0267	–	–
A'	3.79	0.0029	3.64	0.0027	3.44	0.0017	3.78	0.0028	–	–
A"	3.99	0.0030	3.84	0.0028	3.53	0.0042	3.99	0.0027	–	–
A'	4.02	0.0252	3.91	0.0180	3.63	0.0147	4.01	0.0236	–	–
A"	4.03	0.0014	3.86	0.0014	3.54	0.0000	4.03	0.0014	–	–
A'	4.11	0.0005	3.99	0.0031	3.75	0.0052	4.09	0.0002	–	–
A"	4.31	0.0001	4.19	0.0000	3.90	0.0002	4.32	0.0000	–	–
A'	4.35	0.0000	4.23	0.0008	3.91	0.0001	4.35	0.0003	–	–
A'	4.36	0.0008	4.25	0.0001	3.97	0.0002	4.36	0.0006	–	–
A"	4.39	0.0000	4.26	0.0000	3.95	0.0001	4.40	0.0000	–	–
A"	4.41	0.0001	4.29	0.0002	3.98	0.0001	4.42	0.0001	–	–
A"	4.47	0.0000	4.32	0.0000	4.02	0.0000	4.48	0.0000	–	–
A"	5.67	0.0010	5.48	0.0008	5.11	0.0007	5.66	0.0009	–	–
A'	5.70	0.0012	5.52	0.0023	5.06	0.0015	5.70	0.0011	–	–
A"	5.76	0.0000	5.57	0.0001	5.19	0.0000	5.75	0.0000	–	–
A'	5.77	0.0053	5.55	0.0066	5.16	0.0019	5.77	0.0055	–	–
A'	5.78	0.0001	5.56	0.0004	5.16	0.0046	5.78	0.0001	–	–
A'	5.85	0.0029	5.64	0.0024	5.21	0.0019	5.86	0.0027	–	–
A'	6.07	0.0049	5.77	0.0051	5.39	0.0044	6.05	0.0041	–	–
A'	6.18	0.0735	5.90	0.0653	5.51	0.0642	6.16	0.0677	–	–

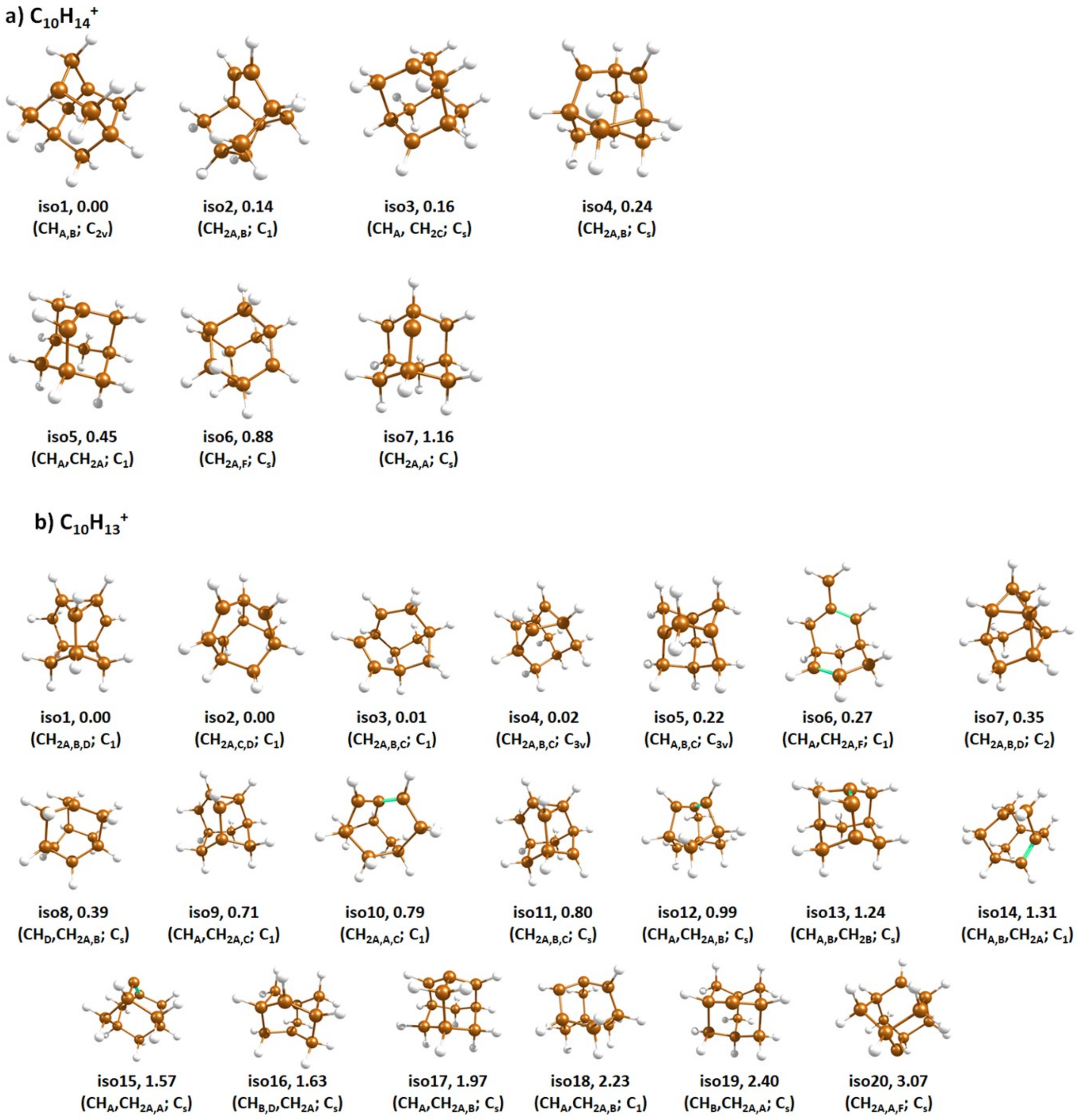

Figure S1 – All isomers located for a) $C_{10}H_{14}^+$ and b) $C_{10}H_{13}^+$ as optimized at the B3LYP+D3/aug-cc-pVDZ level of theory along with the relative energy in eV and the respective symmetry point group. Hydrogens removed from CH and CH_2 groups of $(Adam)^+$ are denoted according to atom nomenclature shown in the manuscript.

Cartesian coordinates of isomers optimized at the B3LYP+D3/aug-cc-pVDZ level (in Å) along with zero-point corrected electronic energies (in Hartree)

(Adam)+
E = -390.222201
C -0.000000 1.452709 -0.553890
C -1.267842 0.731989 -1.003864
H -0.000000 2.508488 -0.856128
H -2.167697 1.251520 -0.648487
H -1.319074 0.761567 -2.108228
C -1.258083 -0.726354 -0.553890
H -2.172414 -1.254244 -0.856128
C -0.000000 -1.463977 -1.003864
C -1.264608 -0.730122 1.059172
H -0.000000 -2.503041 -0.648487
H -0.000000 -1.523135 -2.108228
C 1.258083 -0.726354 -0.553890
H -1.279839 -1.771178 1.401427
C 0.000000 0.000000 1.467714
H -2.173804 -0.222784 1.401427
H 2.172414 -1.254244 -0.856128
C 1.267842 0.731989 -1.003864
H 2.167697 1.251520 -0.648487
H 1.319074 0.761567 -2.108228
H 0.000000 0.000000 2.598167
C 1.264608 -0.730122 1.059172
C 0.000000 1.460243 1.059172
H 2.173804 -0.222784 1.401427
H 1.279839 -1.771178 1.401427
H 0.893965 1.993962 1.401427
H -0.893965 1.993962 1.401427

(Adam)2+
E = -780.792836
C 1.148714 -4.066224 0.000000
C 1.752557 -2.611838 0.000000
H 2.012504 -4.751006 0.000000
H 2.378172 -2.469275 0.890196
H 2.378172 -2.469275 -0.890196
C 0.554166 -1.646289 0.000000
H 1.005380 -0.617700 0.000000
C -0.287645 -1.816075 -1.259045
C -0.287645 -1.816075 1.259045
H -1.121812 -1.099863 -1.280486
H 0.318330 -1.657266 -2.160446
C -0.879439 -3.266948 -1.258900
H -1.121812 -1.099863 1.280486
C -0.879439 -3.266948 1.258900
H 0.318330 -1.657266 2.160446
H -1.491102 -3.395655 -2.161117
C 0.306089 -4.254429 -1.262542
H -0.089312 -5.282902 -1.298485
H 0.921175 -4.118339 -2.162853
H -1.491102 -3.395655 2.161117
C -1.735404 -3.458177 0.000000
C 0.306089 -4.254429 1.262542
H -2.178617 -4.466615 0.000000
H -2.580102 -2.752894 0.000000
H -0.089312 -5.282902 1.298485
H 0.921175 -4.118339 2.162853
C 0.904980 3.094747 1.259322
C 1.777822 3.173052 0.000000
H 1.527058 3.151238 2.161838
H 2.370228 4.099061 0.000000
H 2.504964 2.343459 0.000000
C 0.904980 3.094747 -1.259322
H 1.527058 3.151238 -2.161838
C 0.073587 1.798775 -1.256423
C -0.096824 4.302342 -1.261842
H -0.544032 1.723489 -2.161773
H 0.770729 0.939511 -1.274925
C -0.791455 1.727266 0.000000
H -0.720293 4.265547 -2.164948
C -0.954682 4.219657 0.000000
H 0.482280 5.235971 -1.288728
H -1.408601 0.812667 0.000000
C 0.073587 1.798775 1.256423
H -0.544032 1.723489 2.161773
H 0.770729 0.939511 1.274925
H -1.672090 5.068616 0.000000
C -1.799209 2.933454 0.000000
C -0.096824 4.302342 1.261842
H -2.440036 2.876571 0.889266
H -2.440036 2.876571 -0.889266
H -0.720293 4.265547 2.164948
H 0.482280 5.235971 1.288728

(Adam)3+
E = -1171.358229
C -0.980104 3.177708 1.483795
C -2.081224 2.614537 0.576451
H -1.208437 2.967115 2.536516
H -2.197155 1.532982 0.738927
H -3.050438 3.064951 0.841376
C -1.756992 2.899868 -0.894602
H -2.538835 2.484790 -1.545038
C -1.605318 4.416073 -1.126195
C -0.398824 2.211749 -1.261327
H -1.372946 4.621828 -2.180384
H -2.564234 4.911228 -0.905636
C -0.507302 4.977469 -0.214332
H -0.160101 2.395097 -2.316480
C 0.679504 2.789666 -0.348849
H -0.504060 1.127506 -1.121121
H -0.380927 6.058525 -0.380154
C -0.824709 4.696469 1.258459
H -0.036228 5.103328 1.907048

H -1.763827 5.196692 1.543280
H 1.662829 2.317619 -0.620162
C 0.854830 4.298227 -0.583197
C 0.380958 2.494190 1.116201
H 1.656287 4.704931 0.047540
H 1.105690 4.505285 -1.631613
H 1.177145 2.880076 1.766015
H 0.289941 1.412715 1.293592
C -1.616896 -1.719308 -0.804189
C -1.386485 -1.474075 0.696913
H -0.755325 -1.359496 -1.383363
H -0.473365 -1.987908 1.030341
H -1.231383 -0.399440 0.884418
C -2.595535 -1.975132 1.504863
H -2.426341 -1.796156 2.576017
C -3.871837 -1.243747 1.042800
C -2.769542 -3.496793 1.260973
H -4.737703 -1.592044 1.623548
H -3.772718 -0.163040 1.234727
C -4.102887 -1.493315 -0.457978
H -3.620585 -3.872635 1.846306
C -3.000139 -3.744536 -0.239693
H -1.872381 -4.032323 1.606014
H -5.015137 -0.975353 -0.788411
C -2.893846 -0.988464 -1.263981
H -3.055565 -1.151128 -2.339110
H -2.773850 0.097139 -1.119487
H -3.127497 -4.826565 -0.412828
C -4.276684 -3.015335 -0.702035
C -1.791445 -3.240236 -1.045456
H -4.461186 -3.203777 -1.769496
H -5.150413 -3.385195 -0.146885
H -1.936911 -3.432015 -2.118136
H -0.877692 -3.770908 -0.737092
C 3.795186 -1.292645 1.509237
C 2.579483 -2.184251 1.215364
H 4.034128 -1.314958 2.580917
H 2.796211 -3.220800 1.516843
H 1.715555 -1.859293 1.813272
C 2.242306 -2.142115 -0.283235
H 1.366918 -2.769773 -0.497229
C 1.901844 -0.670378 -0.678947
C 3.451251 -2.599046 -1.119069
H 1.639579 -0.624472 -1.743954
H 1.033151 -0.340603 -0.098040
C 3.117445 0.197513 -0.378242
H 3.209116 -2.571366 -2.191052
C 4.663961 -1.707309 -0.818767
H 3.690138 -3.645960 -0.873427
H 2.877556 1.257731 -0.671411
C 3.449905 0.181991 1.112243
H 4.303374 0.837174 1.330112
H 2.595211 0.525082 1.712166
H 5.529864 -2.017629 -1.422254
C 4.324478 -0.230190 -1.223619
C 5.007852 -1.745525 0.676070
H 5.190002 0.417102 -1.031831
H 4.092855 -0.183977 -2.295914
H 5.878885 -1.108985 0.886814
H 5.285413 -2.771465 0.966779

C10H15+, iso1
E = -389.665579
C 0.000000 1.441829 1.126583
C 0.000000 1.449208 -0.500943
C -1.267299 0.731675 -0.983058
C -1.255051 -0.724604 -0.500943
C -1.248660 -0.720914 1.126583
C 0.000000 0.000000 1.342566
C -0.000000 -1.463350 -0.983058
C 1.255051 -0.724604 -0.500943
C 1.267299 0.731675 -0.983058
C 1.248660 -0.720914 1.126583
H 0.000000 2.508610 -0.784443
H -2.167826 1.251595 -0.630296
H -1.300301 0.750729 -2.081372
H -2.172520 -1.254305 -0.784443
H -0.000000 -2.503190 -0.630296
H -0.000000 -1.501458 -2.081372
H -1.234813 -1.756803 1.477746
H -2.138842 -0.190978 1.477746
H 2.172520 -1.254305 -0.784443
H 2.167826 1.251595 -0.630296
H 1.300301 0.750729 -2.081372
H 2.138842 -0.190978 1.477746
H 1.234813 -1.756803 1.477746
H 0.904029 1.947780 1.477746
H -0.904029 1.947780 1.477746

C10H15+, iso2
E = -389.648033
C 0.756032 1.037009 -1.238867
C -0.003082 1.505552 0.000000
C 0.756032 1.037009 1.238867
C 0.756032 -0.606309 1.247372
C 1.449928 -0.802733 0.000000
C 0.756032 -0.606309 -1.247372
C -0.711428 -1.087082 1.254954
C -1.436330 -0.570766 -0.000000
C -1.449127 0.970885 -0.000000
C -0.711428 -1.087082 -1.254954
H -0.010535 2.607236 0.000000
H 1.781805 1.423879 1.271387
H 0.263037 1.292866 2.185658
H 1.341032 -0.918893 2.118887
H -0.725848 -2.184174 1.297507
H -1.196526 -0.721055 2.168815
H 2.542531 -0.881751 0.000000
H -2.468230 -0.944280 -0.000000
H -1.983370 1.346247 -0.883243
H -1.983370 1.346247 0.883243
H 1.341032 -0.918893 -2.118887
H -1.196526 -0.721055 -2.168815
H -0.725848 -2.184174 -1.297507
H 0.263037 1.292866 -2.185658
H 1.781805 1.423879 -1.271387

C10H14+, iso1
E = -389.024207
C -1.031048 0.000000 0.833399
C 0.000000 0.000000 1.931604
C 1.031048 -0.000000 0.833399
C 1.247930 -1.260082 0.069949
C 1.247930 1.260082 0.069949
C 0.000000 -1.274487 -0.891707
C 0.000000 1.274487 -0.891707
C -1.247930 -1.260082 0.069949
C -0.000000 -0.000000 -1.761558
C -1.247930 1.260082 0.069949
H -0.000000 0.911735 2.535546
H -0.000000 -0.911735 2.535546
H 2.175467 -1.224230 -0.511587
H 1.250633 -2.154297 0.704581
H 2.175467 1.224230 -0.511587
H 1.250633 2.154297 0.704581
H 0.000000 -2.185795 -1.504892
H -2.175467 -1.224230 -0.511587
H -1.250633 -2.154297 0.704581
H 0.000000 2.185795 -1.504892
H -0.881748 0.000000 -2.416320
H 0.881748 -0.000000 -2.416320
H -2.175467 1.224230 -0.511587
H -1.250633 2.154297 0.704581

C10H14+, iso2
E = -389.019161
C 0.526307 -0.004496 -1.326627
C 0.014225 -1.316973 -0.626499
C -1.346967 -1.315158 -0.116659
C 0.794235 -1.314111 0.833290
C -1.879743 -0.115899 0.387405
C 1.260181 0.141019 0.919952
C -1.078553 1.114949 0.241234
C 1.768230 0.394593 -0.505370
C 0.126654 1.117841 1.284958
C -0.515592 1.121255 -1.207806
H 0.753014 -0.216158 -2.376748
H 0.274734 -2.233675 -1.160544
H -1.898990 -2.250957 -0.006456
H 0.185739 -1.636090 1.688540
H 1.611342 -2.037456 0.717058
H -2.859355 -0.112543 0.869209
H 2.041880 0.217357 1.693068
H 2.072240 1.432436 -0.685760
H 2.627472 -0.250990 -0.729412
H -1.694407 1.996047 0.452716
H 0.513515 2.145777 1.280325
H -0.256138 0.917465 2.293862
H -0.048956 2.092575 -1.412786
H -1.335959 0.998094 -1.926335

C10H14+, iso3
E = -389.018324
C -1.296479 -0.417893 0.000000
C -0.853286 -1.041705 1.237767
C 0.754894 -0.568482 1.241706
C 1.331226 -1.103898 -0.000000
C 0.754894 0.978580 1.262855
C 0.754894 -0.568482 -1.241706
C 0.070675 1.519131 0.000000
C -0.853286 -1.041705 -1.237767
C 0.754894 0.978580 -1.262855
C -1.462032 1.043747 0.000000
H -1.293021 -0.627123 2.149406
H -0.888776 -2.134386 1.241973
H 1.189329 -1.001929 2.148490
H 2.007431 -1.958241 -0.000000
H 1.792948 1.334308 1.307136
H 0.253332 1.339457 2.170669
H 1.189329 -1.001929 -2.148490
H -1.293021 -0.627123 -2.149406
H -0.888776 -2.134386 -1.241973
H 0.058398 2.616002 0.000000
H 0.253332 1.339457 -2.170669
H 1.792948 1.334308 -1.307136
H -1.955905 1.427170 -0.898890
H -1.955905 1.427170 0.898890

C10H14+, iso4
E = -389.015224
C -0.860951 -0.185004 1.240650
C -0.466998 1.227599 0.967699
C 0.638533 1.508210 -0.000000
C 1.791659 0.487418 0.000000
C -0.466998 1.227599 -0.967699
C 1.216135 -0.934312 0.000000
C -0.860951 -0.185004 -1.240650
C 0.367451 -1.135935 1.269910
C 0.367451 -1.135935 -1.269910
C -1.743471 -0.549226 -0.000000
H -1.450784 -0.247878 2.161960
H -1.037318 2.059308 1.385411
H 0.937153 2.560857 -0.000000
H 2.411760 0.671831 -0.886664
H 2.411760 0.671832 0.886664
H -1.037318 2.059308 -1.385411
H 2.043778 -1.656468 0.000000
H 0.001464 -2.168465 1.340022
H 0.968706 -0.941693 2.167712
H -1.450783 -0.247878 -2.161960
H 0.001465 -2.168465 -1.340022
H 0.968706 -0.941693 -2.167712
H -1.979722 -1.622691 -0.000000
H -2.680030 0.019624 -0.000000

C10H14+, iso5
E = -389.007546
C 0.421019 -0.529173 1.198779
C 0.442108 0.868046 1.453122
C -0.170294 1.556186 0.237638
C 0.908277 1.210385 -0.860640
C -1.524148 0.936839 -0.155651

C 1.024067 -0.312359 -1.024693
C -1.378509 -0.581159 -0.344097
C 1.543347 -0.994527 0.379626
C -0.328755 -0.925099 -1.411162
C -0.881876 -1.204396 1.042696
H 1.270846 1.351069 1.975448
H -0.237293 2.639031 0.392688
H 0.581422 1.661688 -1.807706
H 1.878547 1.657102 -0.608163
H -1.865804 1.397121 -1.092665
H -2.280376 1.162448 0.606704
H 1.809040 -0.569466 -1.747023
H 1.609870 -2.080532 0.257872
H 2.512660 -0.552748 0.630777
H -2.346894 -1.045025 -0.569916
H -0.242214 -2.013056 -1.533052
H -0.643757 -0.520503 -2.383263
H -0.783338 -2.290885 0.952517
H -1.594120 -0.944710 1.832070

C10H14+, iso6
E = -388.991981
C 1.000302 0.000006 1.156936
C -0.413843 -0.000008 1.663031
C -1.457833 -0.000014 0.585309
C -1.269376 1.257319 -0.302011
C -1.269352 -1.257340 -0.302017
C 0.143227 1.247605 -0.923975
C 0.143252 -1.247598 -0.923981
C 1.254669 1.241757 0.286529
C 0.530155 0.000010 -1.533019
C 1.254692 -1.241737 0.286524
H 1.725691 0.000011 1.984167
H -0.651646 -0.000009 2.723028
H -2.467384 -0.000026 1.011828
H -2.015046 1.283218 -1.108170
H -1.390705 2.173127 0.288789
H -2.015023 -1.283251 -1.108175
H -1.390664 -2.173152 0.288781
H 0.367025 2.118790 -1.549246
H 2.274278 1.264518 -0.118664
H 1.065605 2.189408 0.806200
H 0.367062 -2.118775 -1.549258
H 1.295501 0.000019 -2.316748
H 2.274302 -1.264477 -0.118667
H 1.065645 -2.189394 0.806189

C10H14+, iso7
E = -388.981729
C 0.804725 -0.602100 1.261842
C 1.391926 -0.880976 -0.000000
C 0.804725 -0.602100 -1.261842
C -0.693205 -1.098968 -1.256346
C 0.804725 1.021198 -1.245384
C -1.397655 -0.566725 0.000000
C 0.056310 1.497702 0.000000
C -0.693205 -1.098968 1.256346
C -1.394594 0.975450 0.000000
C 0.804725 1.021198 1.245384
H 1.375580 -0.956499 2.128454
H 1.375580 -0.956499 -2.128454
H -1.157421 -0.722319 -2.177394
H -0.706714 -2.194821 -1.298148
H 0.304131 1.298085 -2.182428
H 1.835587 1.391319 -1.282263
H -2.434601 -0.931067 0.000000
H -1.157421 -0.722319 2.177394
H -0.706714 -2.194821 1.298148
H 0.055254 2.598528 0.000000
H -1.926920 1.353374 0.883173
H -1.926920 1.353374 -0.883173
H 0.304131 1.298085 2.182428
H 1.835587 1.391319 1.282263

C10H13+, iso1
E = -388.438261
C 0.936204 0.097358 1.154587
C -0.593606 0.105324 1.332146
C -1.465851 -0.650714 0.450219
C -1.460666 0.936886 0.189666
C -0.875858 -1.421238 -0.718097
C -0.669752 1.342379 -0.869161
C 0.561427 -0.929435 -0.937475
C 1.390000 1.174821 0.157627
C 0.596856 0.649320 -1.132891
C 1.332554 -1.172354 0.380521
H 1.405328 0.222170 2.137793
H -0.983977 0.383507 2.311699
H -2.442629 -0.955889 0.827703
H -2.317779 1.513463 0.525906
H -1.511576 -1.301695 -1.604476
H -0.863829 -2.490612 -0.468334
H -0.920728 2.250899 -1.422577
H 2.467204 1.138165 -0.042424
H 1.133247 2.198024 0.457319
H 1.014156 -1.414393 -1.807538
H 1.064516 0.970593 -2.066748
H 2.417534 -1.207019 0.231265
H 1.030692 -2.101300 0.877561

C10H13+, iso2
E = -388.438241
C -0.885555 0.085544 1.190188
C -0.185873 -1.238328 0.894982
C 1.300379 -1.217632 -0.028507
C -0.148793 -1.445383 -0.545096
C 1.819612 0.051537 0.055246
C -0.881680 -0.221211 -1.168775
C 0.934615 1.221373 -0.142708
C -1.851037 0.151527 -0.022858
C 0.067309 0.964420 -1.410804
C 0.057845 1.308046 1.155291
H -1.382686 0.028928 2.166322
H -0.074163 -2.028974 1.636114
H 1.920605 -2.094853 0.142616
H -0.263622 -2.438784 -0.979958

H 2.876586 0.186054 0.299413
H -1.384679 -0.511339 -2.096947
H -2.313388 1.137684 -0.147544
H -2.652284 -0.592505 0.075540
H 1.528128 2.137122 -0.240114
H -0.516959 1.865719 -1.635391
H 0.725544 0.779861 -2.269731
H -0.522877 2.238815 1.141162
H 0.698866 1.332915 2.046759

C10H13+, iso3
E = -388.437790
C 0.879791 1.342718 0.231359
C 1.083272 0.575439 -0.965586
C -0.074005 -0.392198 -1.277753
C -0.045644 -1.440823 -0.108853
C -1.420210 0.340049 -1.099808
C 1.261597 -1.244885 0.569161
C -1.512853 0.417848 0.436980
C 1.896265 -0.074204 0.259233
C -1.220789 -1.036173 0.850761
C -0.438546 1.400526 0.963071
H 1.592777 2.135713 0.464271
H 1.737549 0.946491 -1.755681
H 0.062795 -0.853758 -2.259734
H -0.145735 -2.477323 -0.453100
H -2.241534 -0.262497 -1.507096
H -1.443622 1.318954 -1.595572
H 1.695252 -1.994068 1.236023
H 2.915124 0.181859 0.534891
H -2.495718 0.747048 0.792418
H -0.974104 -1.149577 1.914707
H -2.086909 -1.674490 0.642456
H -0.288597 1.316424 2.047633
H -0.780542 2.435435 0.787393

C10H13+, iso4
E = -388.437458
C 0.000000 1.485355 -0.482539
C -0.929274 0.536517 -1.178637
C -1.286355 -0.742678 -0.482539
C -0.000000 -1.073034 -1.178637
C -1.261885 -0.728549 1.044016
C 1.286355 -0.742678 -0.482539
C 0.000000 0.000000 1.550687
C 0.929274 0.536517 -1.178637
C 1.261885 -0.728549 1.044016
C 0.000000 1.457099 1.044016
H 0.000000 2.476480 -0.939420
H -1.337645 0.772290 -2.158955
H -2.144695 -1.238240 -0.939420
H -0.000000 -1.544579 -2.158955
H -1.284429 -1.763610 1.408879
H -2.169546 -0.230543 1.408879
H 2.144695 -1.238240 -0.939420
H 1.337645 0.772290 -2.158955
H 0.000000 0.000000 2.646603
H 2.169546 -0.230543 1.408879
H 1.284429 -1.763610 1.408879
H 0.885116 1.994153 1.408879
H -0.885116 1.994153 1.408879

C10H13+, iso5
E = -388.430317
C -0.000000 1.105975 -0.482377
C -1.290949 0.745330 -1.156361
C -0.957802 -0.552987 -0.482377
C -0.000000 -1.490659 -1.156361
C -1.225952 -0.707804 1.004681
C 0.957802 -0.552987 -0.482377
C 0.000000 -0.000000 1.651132
C 1.290949 0.745330 -1.156361
C 1.225952 -0.707804 1.004681
C 0.000000 1.415607 1.004681
H -2.162755 1.248667 -0.738100
H -1.287055 0.743081 -2.248377
H -0.000000 -2.497334 -0.738100
H -0.000000 -1.486163 -2.248377
H -1.279695 -1.765461 1.286224
H -2.168782 -0.225518 1.286224
H 2.162755 1.248667 -0.738100
H 1.287055 0.743081 -2.248377
H 0.000000 -0.000000 2.748308
H 2.168782 -0.225518 1.286224
H 1.279695 -1.765461 1.286224
H 0.889087 1.990979 1.286224
H -0.889087 1.990979 1.286224

C10H13+, iso6
E = -388.428382
C -1.573579 0.151590 0.074692
C -0.722711 1.224627 -0.282366
C 0.607217 1.044163 -0.850180
C 1.612150 1.243734 0.378781
C 0.814851 -0.357302 -1.444699
C 1.678868 0.007743 1.234867
C 0.376305 -1.390611 -0.394126
C -2.780480 0.496464 0.630223
C 1.123765 -1.164510 0.906270
C -1.151315 -1.284088 -0.176236
H -1.064038 2.244437 -0.078173
H 0.822108 1.864813 -1.550000
H 2.587008 1.477595 -0.075847
H 1.333539 2.122007 0.977828
H 1.873822 -0.489414 -1.695614
H 0.236335 -0.466245 -2.371702
H 2.230458 0.109421 2.171821
H -3.510218 -0.260571 0.925668
H -3.056760 1.539554 0.794415
H 0.595182 -2.402303 -0.759235
H 1.198209 -2.008529 1.593825
H -1.470944 -1.907705 0.668537
H -1.685128 -1.653919 -1.064878

C10H13+, iso7
E = -388.425483

C 0.867016 0.787872 -0.901212
C 0.089142 -0.484895 -1.314733
C -1.266702 -0.734273 -0.878281
C 0.000007 -1.399255 0.000008
C -2.002182 0.207821 0.046167
C 1.266703 -0.734249 0.878298
C -0.867019 0.787892 0.901197
C 2.002182 0.207825 -0.046167
C -0.089148 -0.484870 1.314736
C -0.000002 1.691504 -0.000018
H 1.229738 1.318449 -1.787768
H 0.455365 -1.034243 -2.182391
H -1.819555 -1.528250 -1.383861
H 0.000013 -2.484678 0.000020
H -2.722337 -0.364563 0.644911
H -2.560807 0.970805 -0.512940
H 1.819557 -1.528210 1.383901
H 2.560801 0.970820 0.512930
H 2.722343 -0.364569 -0.644895
H -1.229743 1.318488 1.787741
H -0.455356 -1.034181 2.182424
H 0.650868 2.343956 0.594743
H -0.650872 2.343945 -0.594790

C10H13+, iso8
E = -388.423981
C -0.661753 -1.174140 0.000006
C -1.896785 -0.394734 -0.000001
C -1.294144 1.061287 -0.000009
C -0.039203 1.339818 0.777840
C -0.039197 1.339807 -0.777854
C 0.878332 0.175751 1.178749
C 0.878336 0.175735 -1.178750
C 0.066445 -1.248456 1.257497
C 1.851973 0.065631 0.000002
C 0.066449 -1.248472 -1.257482
H -2.501718 -0.522094 -0.903951
H -2.501721 -0.522082 0.903947
H -2.071961 1.826292 -0.000016
H 0.019317 2.259155 1.357131
H 0.019337 2.259145 -1.357140
H 1.347581 0.305589 2.159419
H 0.819853 -2.039504 1.309816
H -0.577475 -1.245662 2.141106
H 1.347588 0.305563 -2.159420
H 2.478091 -0.835415 0.000009
H 2.515998 0.940855 -0.000003
H 0.819856 -2.039522 -1.309789
H -0.577469 -1.245689 -2.141093

C10H13+, iso9
E = -388.412341
C 0.687636 -0.105517 1.093707
C 0.078167 1.251271 0.938781
C -1.125729 1.251884 0.001755
C 0.266498 1.467226 -0.562074
C -1.867335 -0.049346 -0.197661
C 1.047292 0.202942 -1.034942
C -0.871483 -1.203570 -0.221731
C 1.894363 -0.198586 0.275992
C 0.155131 -1.005666 -1.345237
C -0.127064 -1.264866 1.303118
H 0.178125 1.992461 1.731374
H -1.740367 2.148971 0.071371
H 0.536816 2.432686 -0.983917
H -2.413167 -0.038606 -1.152812
H -2.615905 -0.182258 0.594571
H 1.755508 0.439004 -1.835877
H 2.375996 -1.178381 0.225328
H 2.611711 0.608236 0.464325
H -1.362451 -2.182018 -0.273497
H 0.774964 -1.900663 -1.484278
H -0.393325 -0.846308 -2.284774
H 0.411493 -2.210899 1.397344
H -0.944259 -1.156870 2.020593

C10H13+, iso10
E = -388.409353
C -0.654570 -0.455827 -1.219976
C -0.031776 0.913091 -0.993513
C 1.264642 1.196988 -0.556193
C -0.598895 1.562257 0.104541
C 1.997902 0.088862 0.136084
C -1.292626 0.454940 0.874062
C 0.950034 -0.965545 0.602400
C -1.893543 -0.357965 -0.293838
C -0.149439 -0.389495 1.564852
C 0.307084 -1.542365 -0.670599
H -0.888176 -0.612243 -2.280156
H 1.619761 2.228094 -0.494808
H -0.402993 2.571355 0.467357
H 2.552733 0.530839 0.976483
H 2.746675 -0.375781 -0.524747
H -2.007375 0.819580 1.618838
H -2.259556 -1.340486 0.021981
H -2.720079 0.185446 -0.768265
H 1.493963 -1.743263 1.150937
H -0.653583 -1.235647 2.050483
H 0.322175 0.200562 2.362651
H -0.272175 -2.449510 -0.460601
H 1.075743 -1.808595 -1.407076

C10H13+, iso11
E = -388.408963
C -1.626468 -0.366751 -0.000005
C -0.667268 -1.252652 -0.765588
C 0.705089 -0.686545 -1.175909
C 0.713655 0.955445 -1.251757
C 1.534440 -0.802717 0.000001
C 0.024195 1.504237 0.000005
C 0.705080 -0.686574 1.175906
C -1.466781 1.137223 0.000013
C 0.713661 0.955436 1.251761
C -0.667276 -1.252664 0.765568
H -2.657800 -0.720184 -0.000009
H -1.043304 -2.097089 -1.339227

H 1.130129 -1.045417 -2.117781
H 1.740730 1.325752 -1.359548
H 0.172473 1.174806 -2.180885
H 2.627889 -0.751939 0.000018
H 0.138367 2.598766 0.000011
H -1.959233 1.565345 0.883718
H -1.959246 1.565367 -0.883674
H 1.130110 -1.045441 2.117783
H 0.172491 1.174790 2.180897
H 1.740741 1.325730 1.359539
H -1.043313 -2.097117 1.339188

C10H13+, iso12
E = -388.401877
C 0.758832 1.235967 0.000031
C -0.451094 1.317781 0.777196
C -0.911144 -0.131256 1.208700
C -1.790845 -0.512749 -0.000008
C 0.316582 -1.053474 1.284922
C -0.911150 -0.131191 -1.208703
C 1.149003 -0.937346 -0.000027
C -0.451086 1.317825 -0.777139
C 0.316576 -1.053405 -1.284979
C 1.988393 0.548811 0.000016
H -0.718411 2.186602 1.378695
H -1.453059 -0.094370 2.158293
H -2.042029 -1.581618 -0.000033
H -2.725511 0.060410 0.000011
H -0.003500 -2.098795 1.398757
H 0.934180 -0.815796 2.161030
H -1.453070 -0.094258 -2.158290
H -0.718420 2.186684 -1.378574
H 2.007533 -1.620624 -0.000046
H 0.934170 -0.815681 -2.161078
H -0.003507 -2.098719 -1.398869
H 2.578618 0.590220 -0.918160
H 2.578611 0.590170 0.918199

C10H13+, iso13
E = -388.392757
C -0.741204 -0.501681 1.019777
C -1.677711 -0.773236 0.000000
C -0.741204 -0.501681 -1.019777
C -0.741204 0.988530 -1.297248
C 0.585521 -1.173350 -1.210927
C 0.048161 1.470253 -0.000000
C 1.431359 -0.670474 0.000000
C -0.741204 0.988530 1.297248
C 1.471820 0.872766 -0.000000
C 0.585521 -1.173350 1.210927
H -2.628929 -0.236079 0.000000
H -0.188162 1.254791 -2.203805
H -1.739046 1.441638 -1.322261
H 1.025913 -0.860777 -2.164855
H 0.493029 -2.263934 -1.204106
H 0.076449 2.568392 -0.000000
H -0.188162 1.254791 2.203805
H -1.739046 1.441638 1.322261
H 2.444512 -1.091096 0.000000
H 2.022686 1.228754 0.880708
H 2.022686 1.228754 -0.880708
H 1.025913 -0.860777 2.164855
H 0.493029 -2.263934 1.204106

C10H13+, iso14
E = -388.390121
C -0.730737 0.126274 -1.106880
C 0.600045 0.183105 -1.491484
C 1.543704 0.407651 -0.385815
C 0.902183 1.449702 0.595656
C 1.354462 -1.065804 0.287415
C -0.499598 0.953851 0.702595
C -0.128962 -1.393525 0.582218
C -1.451845 1.291196 -0.329164
C -0.699709 -0.276226 1.524468
C -1.044550 -1.312859 -0.706546
H 0.929412 -0.447289 -2.321965
H 2.595209 0.584369 -0.640484
H 1.421063 1.423303 1.559510
H 0.975462 2.457877 0.174805
H 1.933305 -1.053049 1.221851
H 1.805395 -1.816908 -0.370425
H -2.496777 1.027588 -0.154840
H -1.333208 2.249799 -0.834462
H -0.204618 -2.379857 1.062045
H -1.754257 -0.467812 1.754516
H -0.128255 -0.231150 2.458357
H -2.099808 -1.501377 -0.473847
H -0.712874 -2.025686 -1.469842

C10H13+, iso15
E = -388.380396
C -1.350410 0.396874 0.000000
C -1.391559 -0.918740 0.000000
C -0.120515 -1.628244 -0.000000
C 0.621416 -1.049050 -1.288082
C 0.621416 -1.049050 1.288082
C 0.621416 0.485122 -1.266444
C 0.621416 0.485122 1.266444
C -0.866707 1.007864 -1.303652
C 1.326831 1.014428 0.000000
C -0.866707 1.007864 1.303652
H -0.165740 -2.720594 -0.000000
H 1.643023 -1.448228 -1.239955
H 0.153753 -1.443511 -2.197832
H 1.643023 -1.448228 1.239955
H 0.153753 -1.443511 2.197832
H 1.117009 0.862649 -2.171891
H -0.935693 2.100850 -1.276118
H -1.398346 0.629995 -2.181954
H 1.117009 0.862649 2.171891
H 1.330553 2.112707 0.000000
H 2.376108 0.691234 -0.000000
H -0.935693 2.100850 1.276118
H -1.398346 0.629995 2.181954

C10H13+, iso16
E = -388.378335
C -0.030292 1.127497 -0.000000
C -1.466169 1.476616 -0.000000
C -1.141627 -0.028509 0.000000
C -0.863915 -0.835726 -1.223602
C -0.863915 -0.835726 1.223602
C 0.818535 -0.660412 -1.272650
C 0.818535 -0.660412 1.272650
C 0.818535 0.881609 -1.252810
C 1.137871 -1.289435 0.000000
C 0.818535 0.881609 1.252810
H -1.909420 1.868138 0.915839
H -1.909420 1.868138 -0.915839
H -1.191626 -1.877657 -1.175266
H -1.166964 -0.402849 -2.182172
H -1.191626 -1.877657 1.175266
H -1.166964 -0.402849 2.182172
H 1.235018 -1.172875 -2.146316
H 1.821100 1.313525 -1.154815
H 0.373935 1.276117 -2.173481
H 1.235018 -1.172875 2.146316
H 1.399347 -2.351463 0.000000
H 1.821100 1.313525 1.154815
H 0.373935 1.276117 2.173481

C10H13+, iso17
E = -388.366021
C 0.787034 0.000000 1.226239
C 0.062622 1.201615 1.117565
C -1.019383 1.251328 0.137237
C 0.067256 1.247131 -1.098071
C -1.892458 -0.000000 0.058876
C 0.980623 0.000000 -1.064742
C -1.019383 -1.251328 0.137237
C 1.939256 0.000000 0.240723
C 0.067256 -1.247131 -1.098071
C 0.062622 -1.201615 1.117565
H 0.570727 2.135686 1.378035
H -1.595506 2.181013 0.112312
H -0.567262 1.235876 -1.996350
H 0.642724 2.179963 -1.109863
H -2.468099 -0.000000 -0.874826
H -2.614891 -0.000000 0.885951
H 1.628186 0.000000 -1.951667
H 2.570381 -0.898154 0.262463
H 2.570381 0.898154 0.262463
H -1.595506 -2.181013 0.112312
H 0.642724 -2.179963 -1.109863
H -0.567262 -1.235876 -1.996350
H 0.570727 -2.135686 1.378035

C10H13+, iso18
E = -388.356260
C 0.414828 0.353846 1.390099
C -0.882419 -0.217354 1.376359
C -1.316393 -0.923852 0.201817
C -1.589204 0.606299 -0.480982
C -0.244074 -1.655432 -0.604797
C -0.292092 1.412928 -0.600887
C 1.047503 -0.818711 -0.647116
C 0.508897 1.538007 0.663153
C 0.783579 0.504210 -1.391496
C 1.550192 -0.562318 0.815175
H -1.637909 0.150455 2.076345
H -2.281938 -1.434050 0.262470
H -1.975503 0.319470 -1.468433
H -2.384390 1.142724 0.045572
H -0.616405 -1.851380 -1.618076
H -0.067111 -2.632999 -0.136531
H -0.454073 2.338152 -1.169055
H 1.469299 2.053854 0.581987
H 1.829214 -1.374646 -1.184022
H 1.711598 1.068966 -1.540915
H 0.353379 0.317606 -2.385828
H 2.510559 -0.035647 0.818289
H 1.658377 -1.488242 1.390251

C10H13+, iso19
E = -388.349985
C -0.103349 1.382189 -0.000000
C -0.829198 1.135046 1.215822
C -0.829198 -0.573286 1.216740
C -1.234515 -1.181181 -0.000000
C 0.703492 -1.022742 1.257136
C -0.829198 -0.573286 -1.216740
C 1.407632 -0.490228 0.000000
C -0.829198 1.135046 -1.215822
C 0.703492 -1.022742 -1.257136
C 1.357972 1.090784 0.000000
H -0.324446 1.416098 2.144137
H -1.878845 1.439998 1.212910
H -1.342937 -0.864622 2.139013
H 0.788185 -2.110983 1.336833
H 1.113141 -0.589165 2.178870
H -1.342937 -0.864622 -2.139013
H -0.324446 1.416098 -2.144137
H -1.878845 1.439998 -1.212910
H 2.458186 -0.809720 0.000000
H 1.113141 -0.589165 -2.178870
H 0.788185 -2.110983 -1.336833
H 1.862015 1.474737 -0.893997
H 1.862015 1.474737 0.893997

C10H13+, iso20
E = -388.325371
C 0.065659 1.256910 -0.973218
C 0.139614 -0.012824 -1.607469
C -0.049735 -1.265476 -0.967877
C 1.249034 -1.300957 0.007544
C -1.354334 -1.202715 -0.082079
C 1.223861 -0.049153 0.904356
C -1.298139 0.058755 0.811110
C 1.355365 1.193968 0.003866
C -0.067261 0.007562 1.670262
C -1.243981 1.312409 -0.092787

H 0.163870 2.116482 -1.647458
H -0.023822 -2.137028 -1.633131
H 1.132337 -2.234172 0.572936
H 2.164519 -1.366508 -0.592388
H -1.380775 -2.122823 0.515196
H -2.234214 -1.195817 -0.737597
H 2.090245 -0.086145 1.580041
H 1.317281 2.134384 0.567936
H 2.275395 1.180347 -0.592604
H -2.208204 0.101185 1.423476
H -0.103754 0.007859 2.755989
H -1.193864 2.236724 0.496523
H -2.119505 1.374642 -0.751156
